# High ALDH1A1 expression correlates with poor survival in papillary thyroid carcinoma

**DOI:** 10.1186/1477-7819-12-29

**Published:** 2014-02-03

**Authors:** Yue Xing, Ding-yuan Luo, Miao-yun Long, Shi-lin Zeng, Hong-Hao Li

**Affiliations:** 1 Department of Thyroid Surgery, Sun Yat-Sen Memorial Hospital of Sun Yat-Sen University, Guangzhou, Guangdong Province 510120, China

**Keywords:** ALDH1A1, PTC, Immunohistochemistry

## Abstract

**Background:**

High expression of aldehyde dehydrogenase 1 (ALDH1) has been confirmed in many tumors. This enzyme plays an important role in tumor proliferation, metastasis, and drug resistance. However, in the case of papillary thyroid carcinoma (PTC), the relationship between ALDH1 expression and prognosis remains unknown.

**Method:**

We used tissue microarrays to evaluate ALDH1A1 expression in 247 surgically resected PTC specimens by immunochemistry, and correlated the findings with the clinicopathological parameters.

**Result:**

ALDH1A1 levels were significantly higher than in normal thyroid tissues. Moreover, ALDH1A1 overexpression was significantly associated with extrathyroid extension (*P* = 0.001), pT status (*P* < 0.001), pN status (*P* = 0.016) and TNM stage (*P* < 0.001). The Kaplan-Meier survival analysis suggested that high ALDH1A1 expression reflects a poorer lymph node recurrence-free survival (LN-RFS) and distant recurrence-free survival (DRFS) in PTC patients, as compared with patients having low ALDH1A1 expression. Multivariate analysis confirmed the ALDH1A1 expression was an independent prognostic factor for LN-RFS and DRFS in PTC patients.

**Conclusion:**

In conclusion, high ALDH1A1 expression correlates with poor survival in PTC patients.

## Background

Thyroid carcinoma, a common endocrine malignancy, is the fifth most common cancer in women. In recent years, the incidence of thyroid cancer has shown a rising trend [1]. Papillary thyroid carcinoma (PTC) accounts for more than 80% of thyroid malignancies [2]. One of the challenges surrounding thyroid cancers is that they are difficult to detect in the early stages, because almost no symptoms are present at that time. Generally, PTC displays an indolent course and shows a ten-year survival rate of approximately 80%. However, this does not mean that thyroid carcinoma cannot be progressive [3].

One of the challenges in this field is to identify PTC patients who will exhibit a progressive course of the disease, without extrathyroid extension, distant metastases, and tumor differentiation. Therefore, it is important to find markers that could have prognostic value for thyroid carcinoma. The cancer stem cell hypothesis could explain the occurrence of thyroid cancer and metastasis. According to this hypothesis, cancer stem cells are able to self-renew, differentiate, and mediate the drug resistance of the tumor [4]. Currently, a number of cancer stem cell biomarkers have been identified, including CD133, ALDH1, and IGF [5]. Based on the current study, ALDH1 is emerging as a promising biomarker to predict thyroid cancer invasiveness.

In several human cancers, ALDH1 has been considered an important tumor marker. ALDH1 could be a useful marker for cancer stem cells derived from tumors that do not express high ALDH1 levels [6]. ALDH1 is an enzyme involved in the intracellular synthesis of retinoic acid, and ALDH1A1 is a major ALDH family member that catalyzes the oxidation of retinal to retinoic acid [7]. This function plays an important role in promoting stem cell differentiation [8]. Thyroid cancer cells with high ALDH1 expression levels are tumorigenic and reproduce the phenotypic characteristics of the original tumor [4].

ALDH1 is currently regarded as a marker of thyroid cancer. However, the prognosis and clinical significance of ALDH1A1 is not very clear, particularly in certain histological types of thyroid cancer. In this study, to estimate the predictive value of ALDH1A1 for learning about the aggressive behavior of PTC, we proposed to examine ALDH1A1 expression in PTC tissues and the relationship between ALDH1A1 and known prognostic factors.

## Methods

### Tissue samples and patients

Two hundred and forty-seven PTC patients treated surgically between 2004 and 2010 at the Department of Thyroid Surgery of the Sun Yat-Sen Memorial Hospital of Sun Yat-Sen University were eligible for this study. Before surgery, patients were not receiving any medication. All patients were newly diagnosed cases and underwent total thyroidectomy or lobectomy. After surgery, patients received radioactive iodine ablation according to American Thyroid Association Guidelines [9]. Demographical and clinical data were collected from 247 patients with PTC for gender, age, pT status, pN status, recurrence, extrathyroidal invasion and distant metastatic dissemination. The patients were staged according to the current TNM classification system. The tumor specimens and 50 normal samples from adjacent tissues were obtained as paraffin blocks from the department of pathology of our hospital. All patients were followed up for Tg level and ultrasound per six months. All procedures were in compliance with the patient guidelines and the ethical review process of our institution, and were approved by the Institute Research Medical Ethics Committee of China Medical University.

### Immunohistochemistry

Before dewaxing, the tissue sections were placed in a 60°C baking oven for 20 minutes. Slides were deparaffinized in xylene, rehydrated in a graded alcohol series, and washed in PBS twice for five minutes each time. Sections were heated in 10 mM sodium citrate buffer, pH = 6.0, for 15 minutes in a 95°C water bath for antigen retrieval. Until the buffer cooled down, we performed five-minute PBS washes. Endogenous peroxidase activity was blocked by incubating the sections in 3% H_2_O_2_ at room temperature for ten minutes. Blocking serum was added dropwise at room temperature for 20 minutes to reduce the non-specific background. Anti-ALDH1A1 monoclonal antibodies (ab-134188; 1:100 dilution; Abcam, Cambridge, MA, USA) were added and incubated overnight at 4°C. The sections were washed in PBS three times for two minutes, and subsequently incubated with biotinylated secondary antibody (PK-4001; VECTASTAIN® Elite ABC kits, Vector Labs, USA) for 30 minutes at room temperature. The slides were subsequently incubated with ABC (PK-4001; VECTASTAIN® Elite ABC kits, Vector Labs, USA) for another 30 minutes, washed in PBS, and stained with DAB (3, 3-diaminobenzidine). Finally, the sections were counterstained with Mayer’s hematoxylin, dehydrated, and mounted.

### Scoring of staining

ALDH1A1 mainly distributed in the cytoplasm of thyrocytes. Two investigators who were blinded to the patients’ clinicopathological characteristics scored the sections independently. Immunoreactivity was scored using a semi-quantitative method as follows: (0) < 5% positive epithelial cells; (1) from 5 to 20% positive; (2) from 20 to 50% positive; (3) from 50 to 80% positive; and (4) more than 80% positive thyrocytes. The two scores were multiplied in each case, and the expression was graded as negative (score = 0), low expression (score = 1 to 4), or high (score = 5 to 12).

### Statistical analysis

Data were analyzed by the SPSS software (standard version 16.0, SPSS, Chicago, IL, USA). The ability of ALDH1A1 to predict the correlation between ALDH1A1 expression and clinicopathological features was evaluated using Pearson’s *χ*^2^ test. Lymph node recurrence-free survival (LN-RFS) and distant recurrence-free survival (DRFS) were assessed using the Kaplan-Meier method and compared by the log-rank test. Multivariate survival analysis was performed for all the parameters that were significant in the univariate analysis using the Cox regression model. A *P*-value < 0.05 was considered statistically significant.

## Results

### Patients’ characteristics

All clinical and immunohistochemical data are shown in Table 1. Eighty-nine patients were male with a mean age of 43.9 ± 14.5 years (range, 23 to 72 years) and 158 patients were female with a mean age of 46.3 ± 14.9 years (range, 21 to 83 years). Extrathyroid invasion of the tumor was seen in 73 patients (29.5%) and lymph node metastasis was positive in 76 patients (30.7%). According to the TNM staging system, 78 patients (31.5%) were classified as stage I, 55 (22.3%) as stage II, 69 (27.9%) as stage III, and 45 (18.2%) as stage IV. Median follow-up time was 47 months (range, 15 to 96 months), and during this time, 18.6% of the patients (46 of 247) experienced lymph node recurrences and 9.3% (23 of 247) developed distant organ recurrences.

**Table 1:** Correlation between aldehyde dehydrogenase 1 A1 (ALDH1A1) expression and clinicopathological factors

**Parameter**	**Case**	**ALDH1A1**		***P***
		**Low**	**High**	
Gender				
Female	158	73	85	0.794
Male	89	43	46	
Age				
≤ 45	139	72	67	0.084
> 45	108	44	64	
EI				
Yes	73	28	45	0.001
No	174	108	66	
PT status				
1	85	61	24	< 0.001
2	64	23	41	
3	82	28	54	
4	16	4	12	
ON status				
0	76	27	49	0.016
1	171	89	82	
TNM				
I	78	50	28	< 0.001
II	55	29	26	
III	69	24	45	
IV	45	13	32	

ALDH1A1 (low, the low expression of ALDH1A1; high, the high expression of ALDH1A1); EI, extrathyroid invasion.

### ALDH1A1 expression

**Figure 1:**
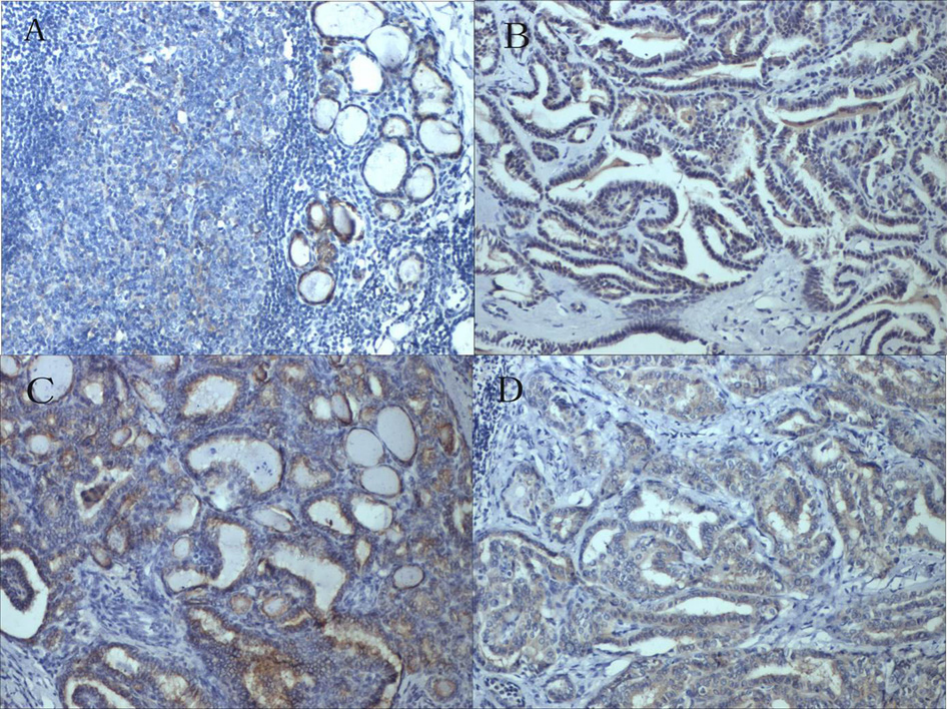
**ALDH1A1 expression by immunohistochemical staining. (A)** Normal thyroid tissue showed low expression of ALDH1A1. **(B)** PTC case demonstrated high expression of ALDH1A1 (×200). **(C)** High expression of ALDH1A1 was detected in a PTC case with extrathyroid invasion (×200). **(D)** High expression of ALDH1A1 was detected in a PTC case with lymph node metastasis (×200).

Immunostaining results were summarized in Table 1. ALDH1A1 expression was significantly higher in malignant tumors than in non-malignant ones. ALDH1A1 expression was present in 78.9% (195/247) of the tumor samples, but only in 32% of the samples from normal and adjacent non-tumor tissues (Figure 1).

### Clinical significance of ALDH1A1

**Figure 2:**
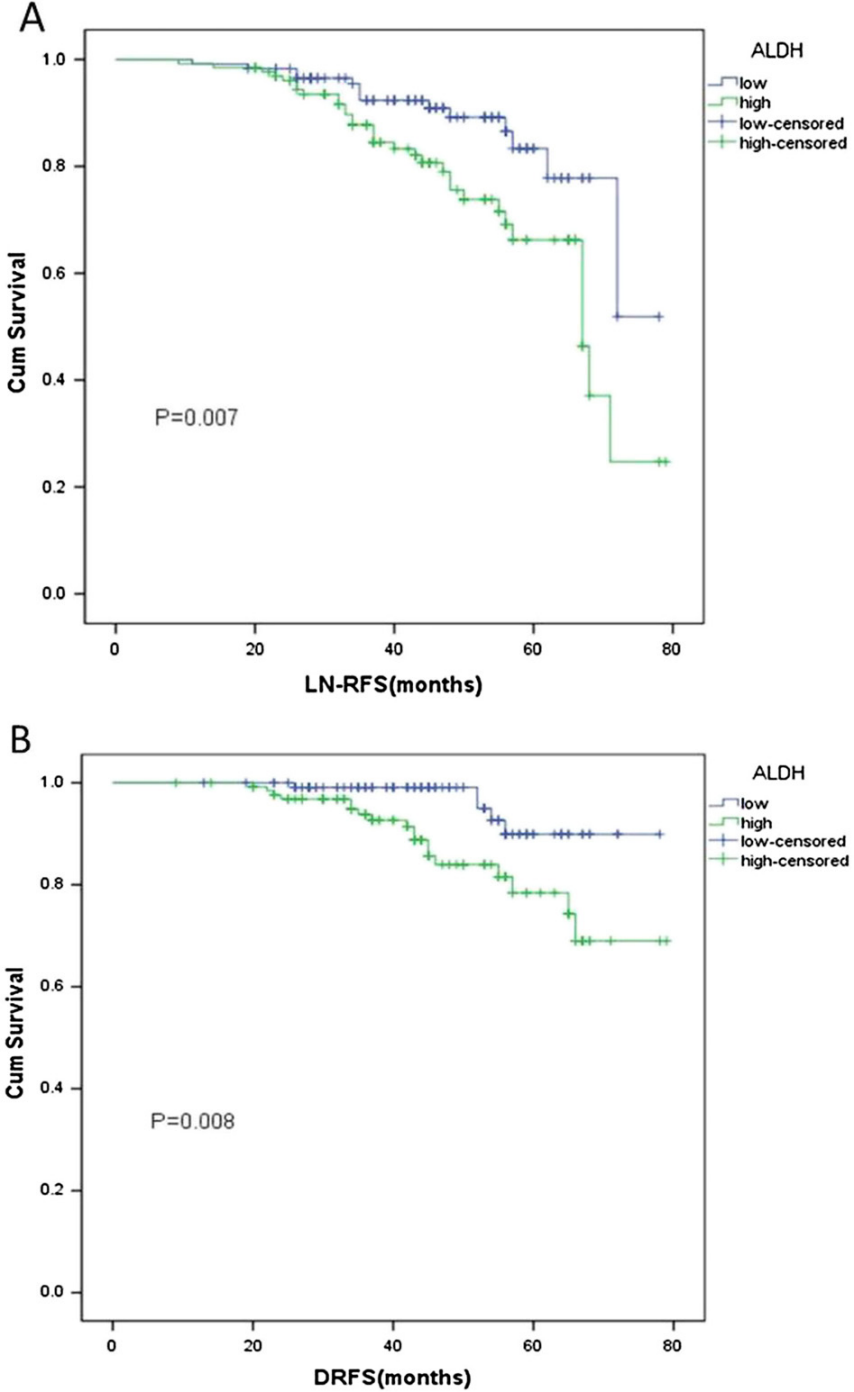
**lymph node recurrence-free survival (LN-RFS) and distant recurrence-free survival (DRFS) curves for papillary thyroid carcinoma (PTC) patients according to their ALDH1A1 expression status. A**, LN-RFS curves: patients with low and high expression levels of ALDH1A1. **B**, DRFS curves: patients with low and high expression levels of ALDH1A1.

As shown in Table 1, a high expression of ALDH1A1 was significantly correlated with extrathyroidal extension (*P* = 0.023), pT status (*P* < 0.001), pN status (*P* = 0.016) and TNM stage (*P* < 0.001). ALDH1A1 expression was not correlated with age or gender. These data indicated that ALDH1A1 expression in tumors can help identify the degree of malignancy of thyroid cancer. The correlation between ALDH1A1 expression levels and thyroid cancer prognosis was analyzed with the Kaplan-Meier method. We observed that ALDH1A1 expression in PTC patients was significantly correlated with LN-RFS (*P* = 0.007) and DR-FS (*P* = 0.008) (Table 2 and Figure 2). When the aforementioned parameters were analyzed in multivariate analysis, the results demonstrated that ALDH1A1 expression was an independent indicator of LN-RFS and DRFS (Table 3).

**Table 2:** Univariate analysis of lymph node recurrence-free survival (LN-RFS) and distant recurrence-free survival (DRFS) in patients with papillary thyroid carcinoma (PTC) (months, mean ± SE)

**Variable**	**Case**	**LN-RFS**	***P***	***DRFS***	***P***
Gender					
Female	158	63.0 ± 2.0	0.073	72.1 ± 1.6	0.362
Male	89	69.3 ± 2.3		72.9 ± 1.8	
Age					
< 45	139	65.3 ± 1.7	0.172	73.4 ± 1.5	0.216
> 45	108	64.9 ± 2.4		71.1 ± 1.9	
EI					
No	174	66.7 ± 1.7	0.036	73.4 ± 1.2	0.028
Yes	73				
PT status					
1	85	60.4 ± 3.4	0.017	68.1 ± 2.9	0.032
2	64	69.4 ± 2.1		74.1 ± 2.8	
3	82				
4	16	66.2 ± 1.8		70.3 ± 2.3	
		65.9 ± 1.3		69.4 ± 1.8	
		60.3 ± 2.5		63.5 ± 2.7	
PN status					
0	171	66.9 ± 1.7	0.028	73.3 ± 1.2	0.046
1	76	60.2 ± 3.2		68.6 ± 2.9	
TNM					
1 to 2	133	68.4 ± 2.2	0.047	75.1 ± 1.6	0.012
3 to 4	114	60.6 ± 1.9		64.7 ± 1.6	
ALDH1A1					
Low	116	69.5 ± 2.1	0.007	75.3 ± 1.2	0.008
High	131	61.6 ± 2.2		69.8 ± 1.9	

ALDH1A1 (low, the low expression of ALDH1A1; high, the high expression of ALDH1A1) EI, extrathyroid invasion.

**Table 3:** Multivariate Cox regression analysis for survival in papillary thyroid carcinoma (PTC)

	**LN-RFS**			**DRFS**		
**Variable**	**RR**	**95% CI**	***P***	**RR**	**95% CI**	***P***
ALDH1A1	2.266	1.207 to 4.254	0.011	3.293	1.216 to 8.917	0.019
T status	1.518	1.292 to 1.932	0.038	2.204	1.134 to 5.276	0.021
N status	2.109	1.381 to 4.170	0.023	2.836	1.285 to 6.305	0.018

ALDH1A1 (low, the low expression of ALDH1A1; high, the high expression of ALDH1A1); CI, confidence interval; RR, relative risk.

## Discussion

As it is widely known, many tumors are not sensitive to chemotherapy, and this is particularly true for thyroid cancer. Many studies demonstrated that ALDH1 overexpression in cancer cell lines leads to resistance to chemotherapy [10,11], which suggests that ALDH1 plays a role in resistance, and indicates that it could provide important insights into understanding the molecular mechanisms of drug resistance in malignancies. In addition, ALDH^high^ cancer stem cells retain their tumor-initiating capacity, and when ALDH^high^ thyroid cancer spheres are topically injected into the mouse thyroid gland, this can lead to the formation of tumors and metastasis [4]. It was revealed that the cancer stem cells that are enriched in ALDH1 can easily lead to cancer recurrences.

According to these findings, the examination of ALDH1A1 expression by immunohistochemistry emerges as an important step towards allowing investigators to identify the patients with a high likelihood of relapse, and this could help design novel therapeutic approaches for PTC patients. The expression of ALDH1A1 by cancer stem cells can have dire consequences, and this is consistent with our findings. Thus, ALDH1A1 may be a new therapeutic target with relevance in thyroid carcinoma.

In this study, we have demonstrated that ALDH1A1 is highly expressed in PTC patients. LN-RFS and DRFS are much shorter in patients with high ALDH1A1 expression, as compared to patients with low ALDH1A1 expression. ALDH1A1 expression was highly correlated with factors that can lead to poor prognosis, such as extrathyroidal extension and lymph node metastasis. Our multivariate analysis confirmed that, in PTC patients, ALDH1A1 overexpression represents the only independent risk factor for LN-RFS and DRFS. Taken together, our results reveal that ALDH1A1 expression levels may be a prognostic indicator in PTC patients.

It is widely accepted that surgical resection could be considered the main therapeutic strategy in patients with PTC. Despite the fact that many patients have the same TNM stage and have undergone the same surgical approach, the prognosis appears to be different in every individual. It is possible that there are some factors, unknown yet, which are involved in shaping prognosis. In our study, high ALDH1A1 protein expression has emerged as an independent prognostic factor for poor LN-RFS and DRFS, as the multivariate survival analysis has demonstrated. Therefore, detecting ALDH1A1 expression might contribute to predicting the prognosis in PTC patients. This factor seems to explain why different prognostic outcomes appear to exist in patients who present the same TNM stages.

ALDH1A1 expression presents clinical significance not only in PTC, but also for other tumors. For example, ALDH1A1 showed high expression in clear cell renal cell carcinoma compared with normal tissues [7]. The ALDH1A1 phenotype is an independent predictor of early tumor relapse that is characteristic of invasive ductal carcinoma [12,13]. Moreover, increased ALDH1A1 expression was associated with enhanced invasiveness in malignancies such as acute myeloid leukemia [14], nasopharyngeal carcinoma [15], bladder cancer [16], and pancreatic cancer [17]. However, in non-small cell lung carcinomas, loss of ALDH1A1 expression was suggested to promote carcinogenesis, particularly in smoking-related adenocarcinomas [18]. These findings may imply that ALDH1A1 overexpression could be involved in accelerating the process of tumorigenesis. In our study, ALDH1A1 has emerged as the main factor associated with poor prognosis.

Our study has certain limitations. Firstly, we did not perform a multi-center study so the number of participants may be insufficient. Secondly, some patients received ^131^I treatment after surgery, which could decrease the rate of recurrence, and may have influenced the results. Thirdly, this is a retrospective study.

## Conclusion

In summary, we demonstrated that ALDH1A1 is overexpressed in PTC. The expression level was correlated with extrathyroidal extension of the tumor and lymph node metastasis. ALDH1A1 may, therefore, be used as an indicator of prognosis in patients with papillary thyroid cancer. However, the mechanisms that underlie this link are not very clear, and further research needs to be performed on this topic.

## Abbreviations

ALDH1: high expression of aldehyde dehydrogenase 1; PTC: papillary thyroid carcinoma; LN-RFS: lymph node recurrence-free survival; DRFS: distant recurrence-free survival.

## Competing interests

The authors declare no potential conflicts of interest.

## Authors’ contributions

YX and D-yL played equally important roles in the development of the experimental protocol. M-yL and S-lZ follow-up cases and interprete the results. Corresponding author is responsible for the tasks of co-ordination arrangements. All authors read and approved the final manuscript.
